# The role of digital technology in surgical home hospital programs

**DOI:** 10.1038/s41746-023-00750-w

**Published:** 2023-02-07

**Authors:** Kavya Pathak, Jayson S. Marwaha, Thomas C. Tsai

**Affiliations:** 1 Harvard Medical School, Boston, MA USA; 2 Department of Surgery, Beth Israel Deaconess Medical Center, Boston, MA USA; 3 Department of Biomedical Informatics, Harvard Medical School, Boston, MA USA; 4 Division of General and Gastrointestinal Surgery, Brigham and Women’s Hospital, Boston, MA USA

**Keywords:** Scientific community, Medical research

## Abstract

Home hospital (HH), a care delivery model of providing hospital-grade care to patients in their homes, has become increasingly common in medical settings, though surgical uptake has been limited. HH programs have been shown to be safe and effective in a variety of medical contexts, with increased usage of this care pathway during the COVID-19 pandemic. Though surgical patients have unique clinical considerations, surgical Home Hospital (SHH) programs may have important benefits for this population. Various technologies exist for the delivery of hospital care in the home, such as clinical risk prediction models and remote patient monitoring platforms. Here, we use institutional experiences at Brigham and Women’s Hospital (BWH) to discuss the utility of technology in enabling SHH programs and highlight current limitations. Additionally, we comment on the importance of data interoperability, access for all patients, and clinical workflow design in successfully implementing SHH programs.

## Introduction

**Fig. 1:**
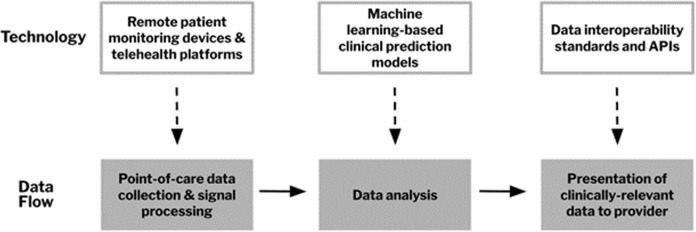
Data flow in a surgical home hospital (SHH) program. Technologies like remote patient monitoring and risk prediction models play roles in data flow.

Home hospital (HH), also known as Hospital-at Home, is a care model of delivering hospital-level care in patients’ homes. It has become increasingly common since the start of the COVID-19 pandemic and the subsequent establishment of the Acute Hospital Care at Home waiver by the Centers for Medicare & Medicaid Services (CMS)^1^. Delivering care at home has been shown to be safe and effective for medical patients, though the introduction of HH programs for surgical patients has been limited^2^. Many components of postoperative management, such as fluid repletion and medication administration, are similar to existing home hospital care for medical patients. This suggests that for surgical patients of lower severity, who do not require mechanical ventilation or repeated imaging, care can be undertaken in the home^3^. For example, home-based management of postoperative surgical site infections could be conducted with intravenous antibiotics, and postoperative vomiting in bariatric patients could be managed at home with fluids and electrolyte repletion^3^. HH programs for surgical patients may provide benefits, including reductions in nosocomial complications and inpatient volume, as well as cost containment^3^. At our institution, Brigham and Women’s Hospital (Boston, MA), SHH has been piloted in bariatric surgery, with ten patients enrolled to date, and additional protocols being developed in plastic surgery, general surgery, and urology. A wide variety of technologies, including remote patient monitoring devices, clinical risk prediction models, and virtual care platforms, exist to facilitate the delivery of care in the home. In this piece, we comment on the utility of these technologies for enabling surgical home hospital (SHH) leveraging our institutional experience. We trace the flow of data from a collection by devices at the point of care to signal processing, analysis, and data integration with health records (Fig. 1), discuss the role technology plays along the care continuum, and propose ways for these steps to be tailored to SHH settings.

## Remote patient monitoring

Remote patient monitoring (RPM) technologies are an important component of HH programs and have the potential to further expand the scope of surgical care delivery in the home. RPM tools record and transmit patient data to providers and can come in the form of wearable or ambient monitoring technologies that collect continuous or intermittent data^4^.

### Wearable devices

Wearable tools include devices such as pulse oximeters and heart rate monitors^5^. Wearable technologies have been used to monitor patients with chronic diseases and patients with COVID receiving care at home^6–8^. Despite its use in medical patients, wearable usage in surgical care remains limited^9,10^.

In our experience at BWH treating bariatric patients in SHH, we use the biovitals^TM^ platform from Biofourmis, which integrates data from wearable devices and uses an AI algorithm to predict health deterioration or improvement^11^. These devices include the Vital Connect VitalPatch, which collects temperature, heart rate, and respiratory rate, a Bluetooth-enabled blood-pressure cuff, and a Bluetooth-enabled pulse oximeter. All patients enrolled in SHH have complied with device usage. Data from these devices are displayed on a secure portal on the Biofourmis website or via a smartphone app accessible to clinicians. Vital sign abnormalities are detected and prompt virtual or in-person visits, depending on severity. The platform also includes a patient-facing interface with daily reminders. Compared to medical complications, surgical complications are relatively infrequent but potentially catastrophic. The ability to detect physiologic signs of stress that serve as early warning signs is critical for the surgical home hospital effort. Real-time temperature, EKG, respiratory rate, blood pressure, and pulse oximetry together provide the advantages of frequent vital checks in a less intrusive manner. Given the array of wearable devices and platforms, surgical home hospitals will need to develop guidelines to assess the types of wearable devices best suited to their patient populations and infrastructure.

### Ambient monitoring tools

Ambient monitoring technologies use modalities like radar or video for contactless monitoring of vital signs and falls^12^. While more nascent, these technologies have been used in the ICU to monitor vital signs and in lower acuity contexts such as at-home elder care and rehabilitation^13–15^. Ambient methods capture patient data by using technologies like cameras, thermal sensors that capture infrared waves, and radio sensors^12^. Sensors have been developed that use radio frequencies to track gait, sleep patterns, and progression of movement disorders^16^. Given the breadth of available technology, there is an opportunity to expand the usage of these tools in surgical care. For example, the usage of ambient monitoring to quantify postoperative mobility in patients after joint replacement may help clinicians identify patients struggling to return to baseline function^17^. Challenges remain in implementing these technologies, as the accuracy of monitoring devices, particularly ambient sensors, may be reduced in complex settings like patients’ homes^12^. While these technologies may function in a more static inpatient environment, the heterogeneity of real-world environments may limit the current utility of these tools.

Though the usage of remote and ambient monitoring tools in surgery has been limited thus far, use cases can be envisioned based on the needs of surgical patients (Table 1). For example, a patient admitted to a home hospital after a sleeve gastrectomy could be provided with wearable sensors to track heart rate and temperature, thus allowing for early detection of postoperative tachycardia and fever, which may indicate a staple line leak. Ambient sensors installed in the patient’s home can provide the care team with valuable information on the patient’s postoperative mobility and ability to perform basic activities.

**Table 1:** Remote patient monitoring technologies and their use cases in surgical patients.

Technology	Frequency	Type of data	Usability	Use case
Wearable heart rate monitor	Every 4 h	Numeric	Intermittent wearable that automatically transmits data	Monitor for postoperative tachycardia
Pulse oximeter	Every 4 h	Numeric	Intermittent wearable that automatically transmits data	Monitor for postoperative pneumonia
Video-enabled smart device for physical exam	Daily	Video	Requires a device with video and audio capabilities	Use for postoperative provider visits
Home-mounted camera for ambulation detection	Continuous	Video	Requires device installed in the home as well as algorithms for data processing	Monitor return to baseline function/ability to do activities of daily living

### Challenges of remote patient monitoring

There are challenges associated with remote patient monitoring that must be addressed as these tools are integrated into SHH programs. Both wearable and ambient technologies present challenges, including signal fidelity, data interoperability, and integration into clinical decision-making. Quality and accuracy of data derived from wearables and ambient monitoring devices are crucial in ensuring the utility of these tools. Determining which vital signs need to be monitored, as well as the appropriate frequency, is also crucial due to concerns about false alarms. Separating true alerts from false alarms could be considered even more critical in HH settings than in traditional care, given the lack of proximity to patients as well as the challenge that alert fatigue presents to patient safety^18^. As HH programs integrate more technologies, often produced by different manufacturers, ensuring data interoperability between devices and data access for remote teams is essential^19^. At BWH, we use a single vendor, Biofourmis, which provides wearable devices and interfaces for remote data access, though data from wearables are not currently integrated into the electronic health record^11^. The approach of using one technology vendor may not be feasible for all hospital systems, and relying on a single vendor could limit the integration of different tools in the future. RPM platforms for surgery must also include the ability to integrate with existing surgical monitoring technologies, such as ViOptix for tissue flap perfusion monitoring^20^. In addition to emphasizing interoperability, increasing the clinical utility of data collected from RPM devices may be accomplished using statistical learning methods. More importantly, HH programs will need new workflows developed by providers to safely integrate patient monitoring tools and data into care delivery. In the BWH experience, the creation of a clinical framework detailing important steps on each postoperative day, including visits from providers and data collection via remote tools and lab testing, has helped to structure the process of postoperative care at home. It is crucial to design workflows that allow remote monitoring data to be accessible to all members of the care team, particularly during rounds and other team-based care events.

## Clinical risk prediction

Clinical risk prediction models are tools that use retrospective observational data and statistical methods—sometimes machine learning-based methods—to predict an individual patient’s likelihood of some clinical outcome^21^. These models have the potential to add value to hospital-at-home programs in two capacities: (1) dynamic prediction of complications to help care teams preempt a patient’s clinical deterioration and mobilize resources accordingly and (2) predict who may benefit from hospital-at-home care versus traditional inpatient care. The use of machine learning in interpreting clinical data can provide unique benefits for patients in SHH. For surgical patients at risk of acute clinical events, it is helpful to have machine learning tools constantly monitoring patient data. Machine learning-based tools that learn with patients’ baseline biometrics can adjust for various activities, such as ambulation in the home, can detect subclinical deviations from this baseline, and can alert a physician when a patient is at elevated risk of an adverse event.

While clinical risk prediction models have been proposed as a potentially useful component of HH programs, they are not yet widely used in these care settings^5^. More broadly, in medicine, few published prediction models have been successfully incorporated into practice^22^. Multiple explanations have been put forth for the lack of these tools’ real-world utility, from their development and validation to the complexity of implementation within existing clinical workflows^23^. In the BWH experience, clinical risk prediction for in-hospital complications, including pneumonia, sepsis, and death, is used to screen patients for surgical home hospital. Patients are deemed high-risk through analysis of demographic factors and comorbidities, and are triaged to inpatient care in the hospital in order to minimize the risk of delays in care escalation or access to additional resources.

When using clinical risk prediction in the care of surgical patients at home, data quality and clinical complications present important considerations. The clinical fidelity of data collected from RPM devices is crucial, as a model’s predictive performance is limited by the reliability of its data inputs. The nature of surgical complications presents another unique challenge. Acute postoperative surgical complications include general complications such as sepsis and myocardial infarction, as well as procedure-specific complications like anastomotic leak after colorectal surgery or duodenal stump leak after gastrectomy—events that are low frequency but potentially catastrophic and time-sensitive when they occur^24,25^. Prediction of rare clinical events presents the risk of alert fatigue and wasted resources if a model flags too many false positives, and unnecessary excess morbidity and mortality for a model with too many false negatives. In addition to accuracy, the risk cutoff threshold used in these models will determine their real-world utility: common risk cutoff selection approaches that equally weight false positives and false negatives may not be ideal in these scenarios. Tools used to predict risk in inpatient settings will likely require careful recalibration to be useful in SHH programs^26^.

Another important challenge lies in translating prediction model outputs into real-world actionable insights. There is a lack of consensus around how prediction models should be incorporated into the clinical decision-making process^27^. Few would argue that these models should fully supplant human judgment - but then how should the provider incorporate the model’s insights into their clinical decision, particularly in cases where the model’s prediction diverges from the provider’s? And what resources should be mobilized or what workflow should be triggered if a patient’s risk of clinical deterioration is high? Most published reports of SHH programs make little use of prediction models in their clinical decision-making^3,5,28^. More research is needed on the optimal integration of clinical prediction models into SHH models.

## Virtual care platforms

Virtual visits between patients and clinicians are critical components of existing home hospital programs and will be crucial to the success of HH within the surgery. Established medical HH programs have created hybrid models which mix in-person and virtual provider visits, and surgical HH programs will need to establish workflows that provide the appropriate schedule of virtual visits based on illness acuity and procedural complexity.^5^ Our model at BWH combines virtual surgeon visits with in-person RN and paramedic visits to provide patients with appropriate observation. Other potential models could include in-person visits from physician assistants, surgicalists, or medical hospitalists in combination with virtual surgeon visits. While performing a physical exam virtually presents some challenges, these issues can be mitigated by having a trained professional (physician assistant or nurse) perform the exam and report findings^29^. Emerging technologies, such as connected auscultation or using built-in smartphone accelerometers to measure abdominal palpation, can allow patients to examine themselves and report findings^30,31^. Additionally, SHH programs must fulfill the need for inpatient consultation by building ways to connect patients with inpatient consult teams. A seamless connection between primary and consulting teams is necessary to ensure appropriate clinical oversight of home hospital patients.

## The path forward

**Fig. 2:**
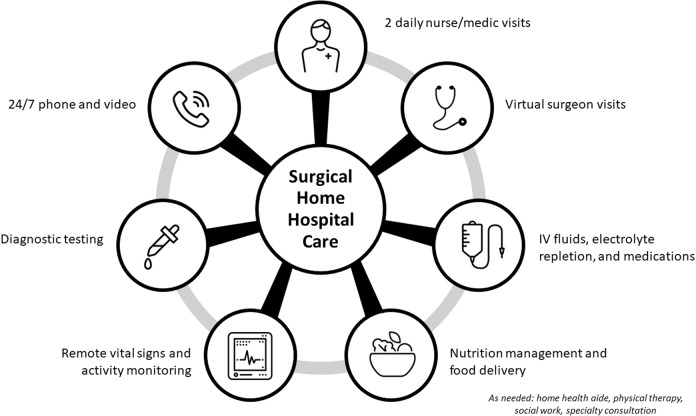
Resources for surgical home hospital care. Multiple types of resources are needed when establishing a surgical home hospital program, including personnel and materials.

While digital technologies are just a few of the many essential elements of an SHH program (Fig. 2), they will likely play an increasingly important role as their capabilities, reliability, and performance improve over time. As SHH programs proliferate, key areas of focus include ensuring data interoperability and equitable access to care services, which will directly impact the success of future SHH programs.

### Ensuring interoperability

The range of patient monitoring devices is increasing, making it crucial that these devices can communicate seamlessly with each other and EHRs^32^. The Office of the National Coordinator for Healthcare IT (ONC) Final Rule, which requires health systems to make Fast Healthcare Interoperability Resources (FHIR)-based application programming interfaces (APIs) available for transmitting and retrieving patient data by December 31, 2022, is an encouraging development that will hopefully make the US healthcare data ecosystem more interoperable and thereby benefit surgical HH programs that depend on communication between RPM devices and EHRs^33^. The Food and Drug Administration (FDA) also supports non-binding consensus standards that encourage medical device interoperability, providing manufacturers with a framework to facilitate device interoperability^34^.

### Secure and equitable access

A final consideration in establishing HH programs within surgery involves ensuring that these programs are designed in a manner that works towards eliminating health disparities instead of widening them. Given disparities in access to surgery and surgical outcomes that racial minorities and patients of lower socioeconomic status experience, it is critical that SHH programs be designed to optimize communication between providers and patients and to account for differences in access to technology^35^. The American Telemedicine Association (ATA) recently put forth a framework for eliminating disparities using telehealth that includes not only connectivity, affordability, and literacy of new digital technologies but also inclusivity and trust. It includes not only clinical care teams but also vendors, policymakers, and community members as key stakeholders^36^. HH programs can similarly be used to tackle disparities in access to quality postoperative care if they are similarly designed with these elements and stakeholders in mind. Additionally, HH programs could potentially decrease disparities in surgical care by increasing provider knowledge of social determinants of health that patients face, information that could be used to connect patients to resources and improve surgical outcomes^37^.

As the technological sophistication of HH programs increases, the tension between access and site-of-care optimization arises. In particular, successful implementation of hospital-at-home programs that depend heavily on technology assumes high levels of patient digital literacy and strong internet connectivity, all of which are not guaranteed, particularly for patients from marginalized groups^38^. 30% of telehealth visits are conducted as audio-only conversations due to infrastructural constraints or technology literacy constraints^39^. These real-world limitations need to be taken into consideration when deploying HH programs as well. Patient-facing technologies should be easy to use for a wide range of literacies and compatible with a wide range of devices. Of particular concern is to ensure that telehealth used in the SHH setting incorporates translation capabilities for patients with limited English language proficiency. Broadband and other IT infrastructure to support these technologies should also be made more widely available and affordable^40^. While some early studies suggest HH can be delivered successfully to patients with lower socioeconomic statuses, surgical HH programs must intentionally design protocols to ensure equal access for all patients^37^. Surgical HH programs designed with equity in mind may not only improve access to care, but may also promote patient inclusivity in research studying post-surgical outcomes^41^. Furthermore, as connected devices proliferate on unsecured networks in non-traditional care settings such as the home, the security of the information being transmitted to and from the patient becomes increasingly relevant. Health systems deploying HH programs should establish strong relationships with their vendors and security standards for the technology that they adopt.

## Conclusion

A successful surgical HH program is one that redesigns clinical workflows instead of entirely replacing them with technology. Current surgical HH programs use many new digital health technologies to enable the delivery of quality care in the home. While there is considerable room for improvement in RPM, clinical risk prediction, and data interoperability, the current functionality of these existing technologies should not be considered a significant barrier to deploying surgical HH programs. The bulk of the innovation in these programs instead lies in developing and operationalizing complex new workflows for providers and staff. As new digital health technologies become more sophisticated, their ability to add clinical value to surgical HH programs will likely increase as well. SHH models need to be rigorously evaluated to ensure that the primary goal of improving patient experience and quality, and not just cost savings, are achieved. The goal of SHH digital health technologies should be to extend, not replace, the clinical presence in patients’ homes. With the thoughtful implementation of remote patient monitoring, SHH care models can do just that: extend clinical expertise into patients’ homes to truly bring new advances in digital health to the patient’s actual bedside.

### Reporting summary

Further information on research design is available in the Nature Research Reporting Summary linked to this article.

## Supplementary information

Reporting Summary

## Data Availability

No data were collected or analyzed for this manuscript.

## Code availability

No code was generated for this manuscript.
